# Midkine Mediates Intercellular Crosstalk between Drug-Resistant and Drug-Sensitive Neuroblastoma Cells *In Vitro* and *In Vivo*

**DOI:** 10.1155/2013/518637

**Published:** 2013-09-03

**Authors:** Fei Chu, Jessica A. Naiditch, Sandra Clark, Yi-Yong Qiu, Xin Zheng, Timothy B. Lautz, Janette L. Holub, Pauline M. Chou, Michael Czurylo, Mary Beth Madonna

**Affiliations:** ^1^Children's Hospital of Chicago Research Center, Chicago, IL 60614, USA; ^2^Department of Surgery, Ann & Robert H. Lurie Children's Hospital of Chicago, Feinberg School of Medicine, Northwestern University, 225 East Chicago Avenue, Box 224, Chicago, IL 60611, USA; ^3^Department of Pathology, Ann & Robert H. Lurie Children's Hospital of Chicago, Feinberg School of Medicine, Northwestern University, 225 East Chicago Avenue, Box 224, Chicago, IL 60611, USA; ^4^Department of Biomedical Engineering, Washington University, St. Louis, MO, USA

## Abstract

Resistance to cytotoxic agents has long been known to be a major limitation in the treatment of human cancers. Although many mechanisms of drug resistance have been identified, chemotherapies targeting known mechanisms have failed to lead to effective reversal of drug resistance, suggesting that alternative mechanisms remain undiscovered. Previous work identified midkine (MK) as a novel putative survival molecule responsible for cytoprotective signaling between drug-resistant and drug-sensitive neuroblastoma, osteosarcoma and breast carcinoma cells *in vitro*. In the present study, we provide further *in vitro* and *in vivo* studies supporting the role of MK in neuroblastoma cytoprotection. MK overexpressing wild type neuroblastoma cells exhibit a cytoprotective effect on wild type cells when grown in a co-culture system, similar to that seen with doxorubicin resistant cells. siRNA knockdown of MK expression in doxorubicin resistant neuroblastoma and osteosarcoma cells ameliorates this protective effect. Overexpression of MK in wild type neuroblastoma cells leads to acquired drug resistance to doxorubicin and to the related drug etoposide. Mouse studies injecting various ratios of doxorubicin resistant or MK transfected cells with GFP transfected wild type cells confirm this cytoprotective effect *in vivo*. These findings provide additional evidence for the existence of intercellular cytoprotective signals mediated by MK which contribute to chemotherapy resistance in neuroblastoma.

## 1. Introduction

Drug resistance poses a major obstacle in the treatment of human cancers. Several mechanisms responsible for resistance to chemotherapy have previously been described. Drug-resistant cells may express one or more energy-dependent transporters like the multidrug resistance gene (*mdr1*) which detect and eject anticancer drugs from cells [1, 2]. Mechanisms including secondary mutations in drug targets or parallel pathways, insensitivity to drug-induced apoptosis [3, 4], and induction of drug-detoxifying proteins also play a role in acquired multidrug resistance (MDR). Numerous attempts have been made to target these pathways and reverse drug resistance. Although some attempts were successful *in vitro*, these strategies were not successfully implemented *in vivo *[5]. These data suggest there are additional, perhaps unknown mechanisms, which need to be identified and targeted for successful reversal or prevention of drug resistance. 

Our laboratory has hypothesized a cytoprotective relationship between drug-resistant and drug-sensitive cells within drug-resistant tumors. We previously demonstrated a mechanism of cytoprotective signaling between drug-resistant and drug-sensitive neuroblastoma, osteosarcoma, and breast cancer cells [6]. A cytokine cDNA array identified midkine (MK) as significantly upregulated in drug-resistant cells and MK consequently was confirmed to play a key role in cytoprotection. 

MK is a retinoic acid-induced neurotrophic factor. It is a small cysteine-rich protein which belongs to the family of heparin-binding proteins and is highly expressed during midgestation in mouse embryogenesis. MK is integral to neuronal development, migration, and neurite outgrowth [7]. MK has been reported to play important roles in the survival, growth, and migration of many cells, which may contribute to oncogenesis and tumor progression. Enriched MK expression has been demonstrated in cancers including Wilms' tumor, neuroblastoma, esophageal, pancreatic, lung, and breast cancers while its expression is usually low in normal human adult tissues [8–10]. In addition, evidence suggests that increased expression of MK is associated with poor prognosis in oral squamous cell carcinoma [11], neuroblastoma [12, 13], and bladder carcinoma [14].

Recently, several other studies have implicated MK in drug resistance. Kang et al. used microarray analysis to assess global gene expression in gastric cancer cell lines with acquired drug resistance to 5-fluorouracil, doxorubicin, and cisplatin (CDDP) [15]. They reported that MK expression was enhanced in all drug-resistant cell line studies, suggesting an important role in drug resistance. Qi et al. reported that MK had cytoprotective activity, preventing CDDP-induced apoptotic cell death through enhancement of *Bcl-2* expression in both murine kidney and cultured Wilms' tumor cells (G401 cells) [16]. These findings support our hypothesis that intercellular cytoprotective signals, such as the one mediated by MK, originate from cells with acquired drug resistance and protect neighboring drug-sensitive cells, thus contributing to chemotherapy resistance.

In the present study, the relationship between MK expression and drug resistance has been further investigated. Special emphasis was placed on determining whether the cytoprotective action of MK observed *in vitro* also occurs *in vivo* using a murine model xenograft.

## 2. Materials and Methods

### 2.1. Reagents

All materials utilized in this study were purchased from the following companies: Dulbecco's modified Eagle's medium (DMEM) (Mediatech, Inc., Pittsburgh, PA); fetal bovine serum (FBS) (HyClone, Logan, UT); doxorubicin; 3-(4,5-dimethyl-2-thiazolyl)2,5-diphenyl tetrazolium bromide (MTT) (Sigma, St Louis, MO); antibodies to midkine (R&D Systems, Minneapolis, MN); antibody to *β*-actin (Sigma, St. Louis, MO); secondary antibodies conjugated to horseradish peroxidase (Promega Corporation, Madison, WI); enhanced chemiluminescence reagents (Pierce Corp., Rockford, IL); Immobilon-P transfer membrane for western blot (Millipore, Bedford, MA); nitrocellulose membranes (Bio-Rad Laboratories, Inc, Hercules, California); TUNEL assay kit (Roche Diagnostics Corporation, Indianapolis, IN).

### 2.2. Cell Culture and Generation of Doxorubicin Resistant Cell Lines

Human neuroblastoma SK-N-SH and osteosarcoma SJSA-1 (OSA) cells were purchased from ATCC (Rockville, MA) and grown in DMEM supplemented with 10% FBS at 37°C in 5% CO_2_ atmosphere. The IC_50_ for SK-N-SH wild type (SK-N-SH WT) cells was determined using the MTT assay. The generation of doxorubicin resistant SK-N-SH cells (SK-N-SH DoxR) was achieved by incubating parental cell lines with incrementally increasing concentrations of doxorubicin ranging from 10^−9^ to 10^−6^ M over a period of 6 months. Cells were deemed resistant after surviving 10 passages in a doxorubicin concentration of 10^−6 ^M, approximately 2 Log above the IC_50_ of the parental cell line. Cells were then continuously selected in DMEM containing doxorubicin 10^−6 ^M. SK-N-SH GFP cells (SK-N-SH GFP-WT) were generated using pLPCX-EGFP retrovirus and selected with puromycin. Human MK overexpressed SK-N-SH cells (SK-N-SH HMK) were derived as previously reported [6].

### 2.3. Co-Culture Cytoprotective Assay

The cytoprotective effect of SK-N-SH DoxR cells on SK-N-SH WT cells was quantitatively evaluated using a co-culture system. The Falcon Cell Culture Insert System (BD Falcon, Franklin Lakes, NJ) was used to allow SK-N-SH WT cells to be co-cultured with either SK-N-SH WT or SK-N-SH DoxR cells. For each co-culture setup, the two cell types were grown in separate compartments without physical contact. Small molecules were able to diffuse through the media between compartments by traversing a 0.4 *μ*m membrane. Cells were seeded and grown for 72 hours prior to being treated with doxorubicin at concentrations of 0, 10^−7^, or 10^−6 ^M for 24 hours. Cell survival was then assayed using trypan blue staining and manual cell counting.

### 2.4. Western Blot Analysis

Whole cell lysates, culture medium, or cell membranes were isolated and subjected to electrophoresis. Whole cell lysates were isolated from cells grown to 80% confluence in 25 cm^2^ flasks. Cells were collected and lysed in a lysis buffer composed of 50 mM HEPES pH 7.4, 150 mM NaCl, 100 mM NaF, 1 mM MgCl_2_, 1.5 mM EGTA, 10% glycerol, 1% Triton X100, 1 *μ*g/mL leupeptin, and 1 mM phenyl-methyl-sulfonyl-fluoride. These whole cell lysates were subjected to sonication. Culture medium was collected from cells grown to 80% confluence in 25 cm^2^ flasks. Initially grown in DMEM supplemented with 10% FBS, cells were then serum starved for 48 hours in 2 mL of low fetal bovine serum culture medium. Cultured medium was then collected and frozen at −20°C prior to being subjected to western blot. Cell membranes were isolated using a well-established protocol [17].

Protein concentrations were determined for whole cell lysates and cell membrane fractions, and equal quantities of protein were separated by electrophoresis on a 4–20% SDS-PAGE gel and transferred to nitrocellulose membranes. The volume of medium loaded on the gel was normalized based on cell count in the flask prior to medium collection. Nitrocellulose membranes were incubated with antibodies for proteins of interest (anti-MK, anti-P-glycoprotein or *β*-actin) and subsequently incubated with appropriate secondary antibody conjugated to horseradish peroxidase. Protein expression was then detected using chemiluminescence.

### 2.5. siRNA Design and Transfection

Human MK siRNA was synthesized by Dharmacon (Lafayette, CO). The nucleotide sequences for the human MK genes were obtained from the NCBI sequence viewer program. The human MK siRNA was based on sequence 229 to 249 and consisted of the following nucleotides: 5′-AAGAAGGAGTTTGGAGCCGAG-3′. On the day before transfection, 3 × 10^5^ cells were seeded into 6-well plates and grown in 2.5 mL of DMEM supplemented with 10% fetal bovine serum. After 24 hours in culture, 25 *μ*L of 20 *μ*M stock solution of siRNA duplexes was transfected into cells using GeneSilencer siRNA transfection reagent according to the manufacturer's protocol (Gene Therapy Systems, San Diego, CA). Cells were maintained in culture for an additional 96 hours before measuring expression of the silenced molecules by western blot and ELISA assay. 

### 2.6. Cell Proliferation Assay

Cell proliferation was assessed using the MTT assay. Cells were seeded in 96 well plates and incubated with logarithmic concentrations of doxorubicin, ranging from 10^−9^ to 10^−5 ^M. Cell proliferation was quantitatively estimated by use of a colorimetric assay using 3-(4,5-dimethylthiazol-2-yl)-2,5-diphenyltetrazolium bromide (MTT). MTT (10 *μ*L of 5 mg/mL solution) was added to each well of the plate and incubated for 4 hours at 37°C. The cells were then solubilized by the addition of 100 *μ*L of 10% SDS/0.01 mmol/L HCl and incubated for 15 hours at 37°C. The absorbance of each well was determined in an ELISA plate reader using an activation wavelength of 570 nm and a reference wavelength of 650 nm. Cell viability in the presence of different doses of doxorubicin was determined by comparison with untreated control cells. 

### 2.7. *In Vivo* Studies

The animal protocol used in this study was approved by the Animal Care and Use Committee of the Lurie Children's Hospital of Chicago Research Center (no. 2006-29). Severe combined immunodeficiency (SCID) or nude mice (Charles River Laboratories, Wilmington, MA), approximately 4–6 weeks of age and weighing approximately 30 g received subcutaneous (SC) tumor implants performed using various ratios of GFP expressing SK-N-SH wild type cells (SK-N-SH GFP-WT) and SK-N-SH doxorubicin drug-resistant cells (SK-N-SH DoxR) or SK-N-SH HMK (SK-N-SH HMK) cells with a total of 10^6^ cells in 100 *μ*L per implant. GFP-WT/DoxR implants were performed at ratios as follows: (a) SKN-SH GFP-WT only, (b) 1 : 1 ratio of SK-N-SH GFP-WT and SK-N-SH DoxR, (c) 1 : 2 ratio of SK-N-SH GFP-WT and SK-N-SH DoxR, and (d) 1 : 4 ratio of SK-N-SH GFP-WT and SK-N-SH DoxR. GFP-WT/HMK implants were performed at ratios as follows: (a) SKN-SH GFP-WT only, (b) 1 : 1 ratio of SK-N-SH GFP-WT and SK-N-SH HMK, (c) 1 : 2 ratio of SK-N-SH GFP-WT and SK-N-SH HMK, and (d) 1 : 4 ratio of SK-N-SH GFP-WT and SK-N-SH HMK. 

When tumors were palpable, the animals were challenged with doxorubicin (2.5 mg/kg). A total of three doxorubicin injections, each separated by 3 days, were performed. Mice were weighed and checked for clinical signs of drug toxicity and lethality. Tumor measurements were made with a caliper three times weekly for 3 to 4 weeks and converted to tumor volume by using the formula *W* × *L* ^2^/2. Tumor growth curves were generated. At 4 weeks after implant, the mice were euthanized and the tumor area was measured after aseptic excision from the host. Tissue specimens were processed and stained with hematoxylin and eosin to assess tumor morphology and GFP expression. 

### 2.8. TUNEL Assay

Terminal deoxynucleotidyl transferase dUTP nick end labeling (TUNEL) allows detection of genomic DNA cleavage which occurs during apoptosis by incorporating fluorescein labels in nucleotide polymers. TUNEL was used to detect apoptotic cells amongst SK-N-SH GFP-WT cells grown in co-culture with SK-N-SH WT, DoxR, or HMK cells. Labeling was quantified using fluorescence microscopy (*in situ* Cell Death Detection kit; Roche Molecular Biochemicals, Indianapolis, IN). The total number of GFP-WT cells per high-powered field was counted using the 40x objective. TUNEL-positive nuclei were manually counted using the same power objective.

### 2.9. Immunohistochemistry for Midkine

After obtaining Internal Review Board approval (IRB# 2010-14080), pediatric patients treated for neuroblastoma from January 1999 to December 2008 at a free-standing, tertiary care children's hospital were retrospectively identified. Only patients who completed therapy before December 2008 and who no longer require biopsy tissue for diagnostic purposes were included in the study. Medical records were reviewed for the following clinical features: age at diagnosis, pathology features (favorable versus unfavorable), N-MYC amplification status, tumor stage, and outcome (mortality). Paraffin embedded tissue samples were collected from the pathology bank, and slides were created.

Formalin-fixed paraffin embedded slides were dewaxed in xylene and hydrated through a graded series of alcohols. Endogenous peroxidases were blocked with a 3% hydrogen peroxide treatment; antigen retrieval was performed by boiling for 20 minutes in a 0.01 M sodium citrate (pH 6) solution, and endogenous biotin blocked using Avidin/Biotin Blocking Kit (Vector Labs, SP-2001). Slides were incubated overnight in the primary antibody for MK (1 : 250 dilution). Following incubation with the appropriate biotin-labeled secondary antibodies, the labeled antigens were visualized by streptavidin-biotin (Vectastain Elite ABC kit; Vector Laboratories) followed by ImmunoPure Metal Enhanced DAB Substrate (Thermo Scientific) and counterstained with hematoxylin (Richard-Allen Scientific).

Cytoplasmic and nuclear staining were scored on a scale of none (0), low (1+), medium (2+), and high (3+) on a blinded basis. When both prechemotherapy and postchemotherapy biopsy specimens were available, expression of MK was compared to determine if MK expression changes after treatment in tumor cells which remain viable despite chemotherapy. Expression of MK was also compared between patients by age at diagnosis (<1 year versus >1 year), stage, N-MYC amplification status (amplified versus nonamplified), histology (favorable versus unfavorable), and survival status. 

### 2.10. Statistical Analysis

Data are expressed as means ± SE. Differences in measured variables between the experimental and control groups were assessed using Student's *t*-test. Statistical calculations were performed using the Statview statistical package (Abacus Concepts, Berkeley, CA). *P* < 0.05 was considered statistically significant.

## 3. Results

### 3.1. Protection of Drug-Sensitive Cells by Drug-Resistant Cells in Coculture Is Mediated by Midkine

**Figure 1:**
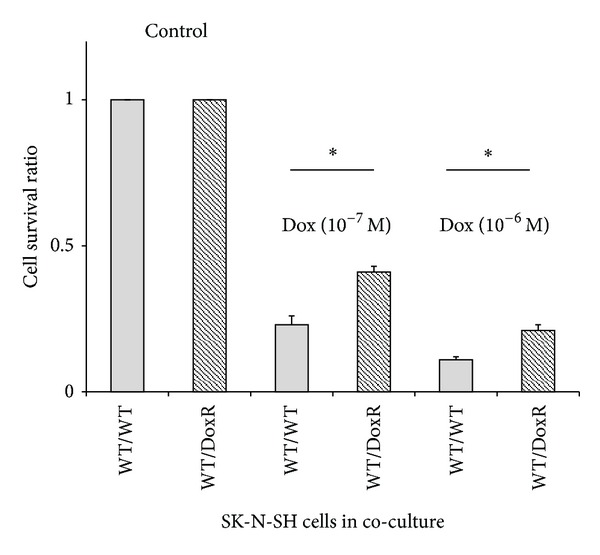
Co-culture effect on cellular response to doxorubicin. Wild type human neuroblastoma (SK-N-SH WT) cells co-cultured with SK-N-SH WT cells (WT/WT) were compared to co-cultures of SK-N-SH WT cells with doxorubicin resistant cells (SK-N-SH DoxR) (WT/DoxR). Co-cultures were treated with and without doxorubicin at 10^−7^ or 10^−6 ^M for 48 hours. Surviving cells were quantified using trypan blue staining. The cell survival ratio represents the number of live WT cells in the drug-treated co-culture divided by the number live cells in the untreated (control), *P < 0.001.

Earlier studies revealed that MK was able to exert a survival function in a variety of cellular systems and against various stimuli, suggesting that it could be associated with drug resistance [6]. Overexpression of MK in doxorubicin-resistant SK-N-SH human neuroblastoma cell lines confirmed this assumption and suggested that it may play a role as a cytoprotective signal between drug-resistant and drug-sensitive cells. We have previously shown that conditioned medium from doxorubicin resistant cells exerts a protective effect on their parental wild type cell line against drug toxicity [6]. Further co-culture experiments were performed to study its cytoprotective role. Cells cannot move between chambers in the co-culture setup; however, small proteins such as MK can freely diffuse between chambers through the co-culture membrane (0.4 *μ*M). Cytoprotective activity was quantitatively determined by comparing the survival of wild type, drug-sensitive neuroblastoma SK-N-SH cells (SK-N-SH WT) co-cultured with either doxorubicin resistant (SK-N-SH DoxR, WT/DoxR) or doxorubicin sensitive (SK-N-SH WT, WT/WT) cells for 72 hours and then treated with doxorubicin at concentrations of 10^−7^ and 10^−6 ^M for 48 hours (Figure 1). The cell survival ratio of SK-N-SH WT cells co-cultured with SK-N-SH WT cells (WT/WT) in this system was 0.23 compared to 0.37 for co-cultures of SK-N-SH WT and SK-N-SH DoxR cells (WT/DoxR) incubated with 10^−7^ M doxorubicin (*P* < 0.001); the cell survival ratio was 0.11 for WT/WT and 0.21 for WT/DoxR cells incubated with 10^−6^ M doxorubicin (*P* < 0.001). Thus, coincubation of drug-sensitive cells with drug-resistant cells was associated with an increase in cell survival ratio significantly above that observed with drug-sensitive cells.

**Figure 2:**
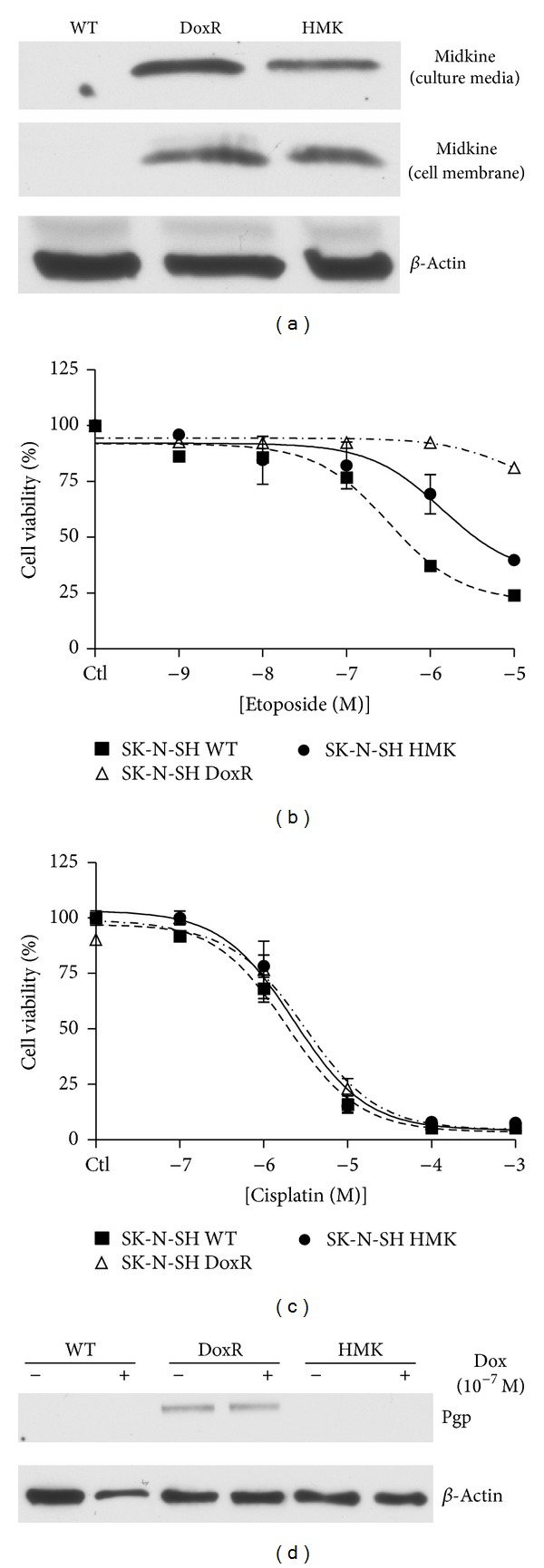
Effect of midkine overexpression on wild type cell survival. A midkine overexpressing SK-N-SH cell line (SK-N-SH HMK) was created as previously described [6]. (a) Midkine expression in the medium of wild type (WT), doxorubicin resistant (DoxR), and HMK cells were analyzed using western blot. SK-N-SH WT, DoxR, and HMK were cultured in 25 cm flasks to 80% confluence. Medium was then exchanged for 2 mL of low fetal bovine serum culture medium and cultured for an additional 48 hours. Cultured medium was then collected and frozen at −80°C prior to being subjected to western blot. The volume of medium loaded on the gel was normalized based on cell count in the flask prior to medium collection. Cell membranes were isolated through a well-established protocol. We have previously demonstrated that midkine transfected cells (HMK) have acquired some doxorubicin resistance [6]. The MTT cell survival assay was performed to determine if SK-H-SH HMK cells have acquired resistance to other chemotherapeutic drugs including (b) etoposide and (c) cisplatin. (d) SK-N-SH WT, DoxR, and HMK cells were treated with or without doxorubicin at 10^−7 ^M for 24 hours prior to collection of cell lysates. Proteins were extracted and probed using western blot for Pgp and *β*-actin.

These findings demonstrate that ligands secreted by doxorubicin resistant human neuroblastoma cells impart a cytoprotective effect on drug-sensitive cells, providing further support for the humoral mediation of resistance to chemotherapy. In order to confirm that MK was the molecule responsible for this protective effect, we created a MK over expressing cell line (SK-N-SH HMK) as previously described [6]. Western blot analysis confirmed that MK was found in the cell membrane fraction and culture medium for these SK-N-SH HMK cells as seen in the SK-N-SH DoxR cells (Figure 2(a)). The MTT cell survival assay previously demonstrated increased survival for a given concentration of doxorubicin for the SK-N-SH HMK cell relative to SK-N-SH WT cell, but not to the extent of SK-N-SH DoxR cells [6]. Here, the MTT assay also demonstrated that the SK-N-SH HMK cell line displayed resistance to etoposide (Figure 2(b)). The SK-N-SH WT, DoxR, and HMK cell lines were equally susceptible to cisplatin (Figure 2(c)).

The *mdr1* gene is known to be an inducible drug resistance gene [18]. To rule out a protective role for *mdr1*, the gene for p-glycoprotein (Pgp) in the SK-N-SH HMK cell line, we compared Pgp expression levels in all aforementioned three cell lines with or without doxorubicin (10^−7 ^M) treatment using western blot analysis.  Figure 2(d) demonstrates that Pgp is expressed only in SK-N-SH DoxR cells and not in SK-N-SH WT or HMK cells either at baseline or when treated with doxorubicin. These data suggest that *mdr1* does not contribute to the self-protective effect demonstrated in SK-N-SH HMK cells.

**Figure 3:**
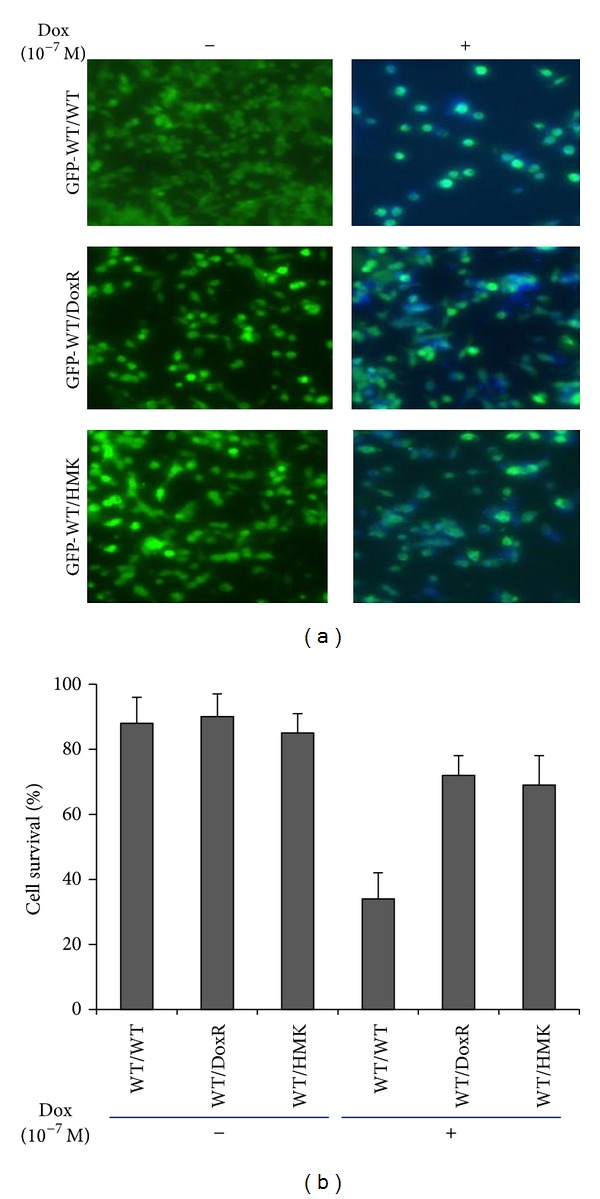
Effect of midkine overexpression on cellular response to doxorubicin in co-culture conditions. (a) GFP-transfected wildtype SK-N-SH cells (GFP-WT) were co-cultured with wild type (WT), doxorubicin resistant (DoxR), or human midkine overexpressing SK-N-SH cells (HMK), incubated with (+) or without (−) doxorubicin at 10^−7 ^M for 48 hours. Photographs were taken using fluorescence microscopy. (b) The viability percentage of fluorescent cells (GFP-WT) after each treatment was determined through Hoechst 33342 staining and cell counting. Data represent an average of three independent determinations +/− SE.

Previous co-culture experiments using GFP-transfected wild type SK-N-SH cells (GFP-WT) co-cultured with either SK-N-SH WT cells or DoxR cells confirmed the cytoprotective effect of SK-N-SH DoxR cells suggesting humoral protection. In order to confirm that MK was the molecule responsible for this effect, these co-culture experiments were repeated using the MK overexpressing cell line (SK-N-SH HMK). Co-cultures were grown using SK-N-SH GFP-WT cells in co-culture with either GFP-WT cells, doxorubicin resistant cells (SK-N-SH DoxR), or human MK overexpressing SK-N-SH cells (SK-N-SH HMK). Cells were grown in co-culture for 48 hours then incubated with (+) or without (−) doxorubicin (10^−7 ^M) for 48 hours. Inserted SK-N-SH WT cells were then stained using Hoechest 33342 to determine cell viability. As seen in Figures 3(a) and 3(b), the GFP cells alone retained their original sensitivity to doxorubicin; however, when co-cultured either with nonfluorescent doxorubicin resistant cells or HMK transfected cells and treated with doxorubicin, the number of viable cells per defined area was higher than that with the drug-sensitive cells alone. These data support the hypothesis that MK is responsible for a humoral cytoprotective effect of drug-resistant cells on nearby drug-sensitive cancer cells.

### 3.2. siRNA to Midkine Abolishes the Protective Effect of Drug-Resistant Cells in Co-Culture with Drug-Sensitive Cells

**Figure 4:**
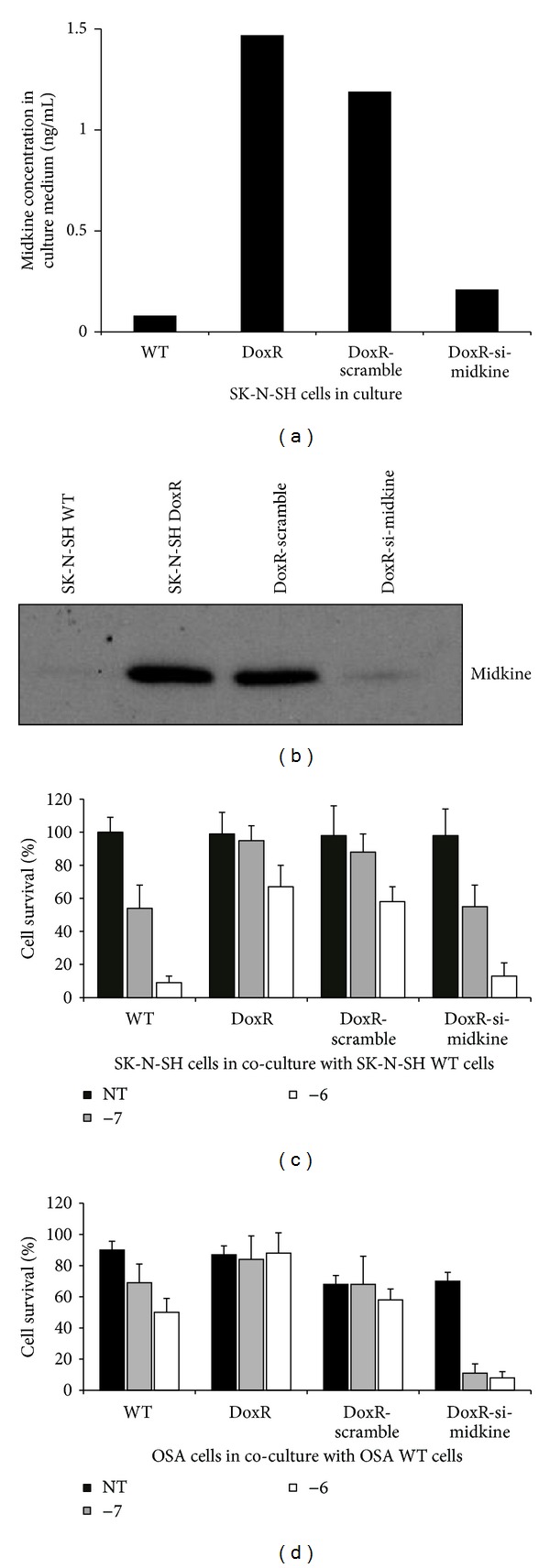
Effect of midkine siRNA on cytoprotection. (a) ELISA assay and (b) western blot were used to confirm decreased expression of midkine in doxorubicin resistant SK-N-SH cells (DoxR) treated with midkine. Culture medium from SK-N-SH wild type (WT), DoxR, DoxR cells treated with scramble sequence siRNA (DoxR-scramble), and DoxR cells treated with siRNA to midkine (DoxR-si-midkine) was harvested after growth for 96 hours. (c) SK-N-SH WT cells were grown in co-culture with WT, DoxR, and DoxR-scramble or DoxR-si-midkine cells. Co-cultures were incubated for 48 hours with or without doxorubicin at 10^−7 ^M and 10^−6 ^M. SK-N-SH WT cell survival was then quantified through cell counting after staining with trypan blue. Data represents the average of 4 experiments +/− SE. (d) OSA WT cells were grown in co-culture with OSA WT, DoxR, and DoxR-scramble or DoxR-si-midkine cells. Co-cultures were incubated for 48 hours with or without doxorubicin at 10^−7 ^M and 10^−6 ^M. OSA WT cell survival was then quantified through cell counting after staining with trypan blue. Data represents the average of 4 experiments +/− SE.

To further investigate the cytoprotective function of MK, we set out to determine if inhibition of MK secretion reversed or reduced the cytoprotective action exerted by doxorubicin resistant neuroblastoma cells upon wild type, drug-sensitive cells. siRNA to MK was used to knock down MK expression in doxorubicin resistant cells. ELISA (Figure 4(a)) and western blot (Figure 4(b)) confirmed decreased expression of MK in doxorubicin resistant cell culture medium when treated with siRNA to MK.

SK-N-SH WT cells (Figure 4(c)) or OSA cells (Figure 4(d)) were grown in co-culture with their respective WT cells, DoxR cells, DoxR cells treated with scramble sequence siRNA, or DoxR cells treated with MK siRNA. Co-cultures were untreated or treated with doxorubicin (10^−7^ or 10^−6^). The results demonstrate that drug-resistant cells transfected with MK siRNA no longer exert a cytoprotective effect. The scramble siRNA exerted a very limited effect on cytoprotection, and only at a doxorubicin concentration of 10^−7 ^M. A parallel study carried out with pleiotrophin siRNA did not affect the cytoprotective activity of doxorubicin resistant cells on wild neuroblastoma cells (data not shown). These data utilizing both a neuroblastoma cell line and an osteosarcoma cell line suggest this phenomenon may be generalized across multiple tumor types. Collectively, these findings support the conclusion that MK plays a dominant role in the cytoprotection induced by drug-resistant cancer cells. 

### 3.3. Midkine Is Partially Responsible for Drug Resistance in Drug-Resistant Cells Themselves

**Figure 5:**
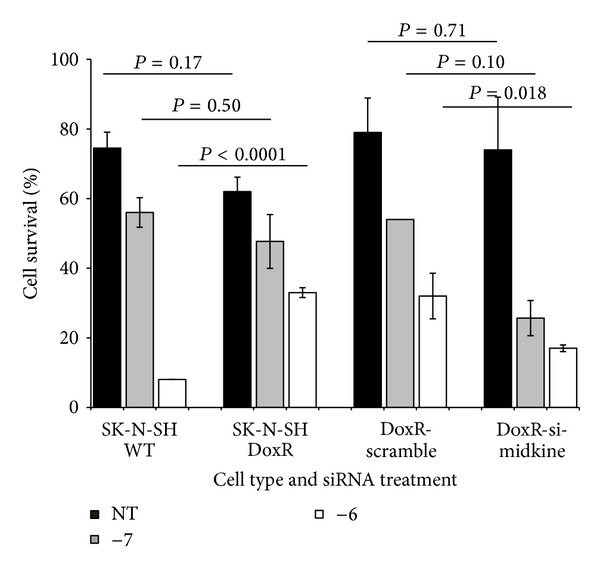
Effect of midkine siRNA on doxorubicin resistant cellular response to doxorubicin. siRNA was used to knock down midkine expression in doxorubicin resistant SK-N-SH cells (DoxR) to determine if loss of midkine expression results in restoration of drug sensitivity in the DoxR cells. Wild type (WT), DoxR, DoxR cells treated with scramble sequence RNA (DoxR-scramble) and SK-N-SH DoxR cells treated with siRNA to midkine (DoxR-si-midkine) were cultured with or without doxorubicin at 10^−7 ^M and 10^−6 ^M for 24 hours. Cell survival was assayed using trypan blue and cell counting. Data represents the average of 4 experiments +/− SE.

We have shown that induced expression of MK in wild type SK-N-SH cells confers a resistance to doxorubicin. We sought to determine if MK plays a self-protective effect in doxorubicin resistant cells themselves. We used MK siRNA to knock down MK expression in doxorubicin resistant (SK-N-SH DoxR) cells. Cells were either untreated or treated with doxorubicin (10^−7^ and 10^−6^ for 24 hr) (Figure 5). When compared with SK-N-SH DoxR cells untreated with siRNA or treated with scramble sequence siRNA, those treated with siRNA to MK had drug sensitivity partially restored. These data suggest MK plays a self-protective role in drug-resistant cells in addition to its humoral protective effect on nearby otherwise drug-sensitive cells.

### 3.4. Doxorubicin Resistant Cells and Midkine Transfected Wild Type Cells Confer Cytoprotection *In Vivo*

**Figure 6:**
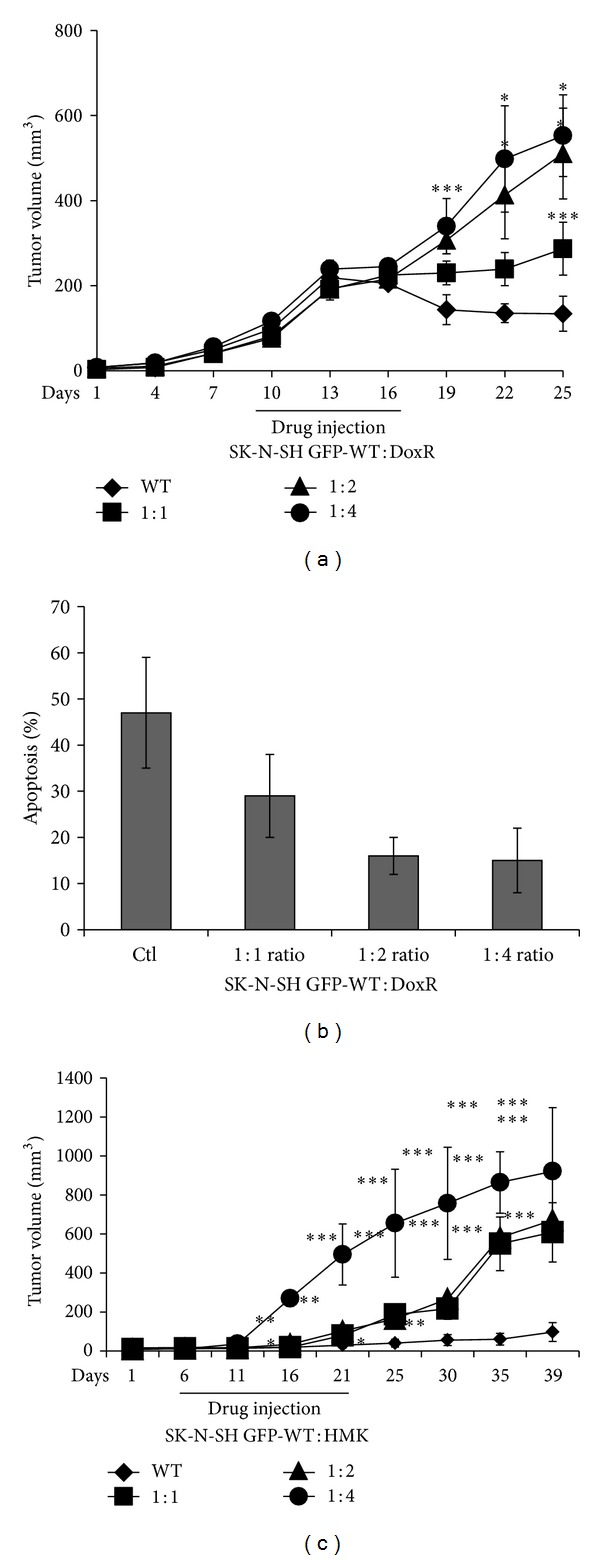
Effect of midkine cytoprotection on growth of wild type human neuroblastoma tumors *in vivo*. Mice were injected with drug-sensitive human neuroblastoma cells (SK-N-SH GFP-WT) and (a) drug-resistant human neuroblastoma cells (SK-N-SH DoxR) or (b) midkine transfected cells (SK-N-SH HMK) at various ratios (GFP-WT : DoxR or GFP-WT : HMK). Once the tumors were palpable, the mice received injections of doxorubicin (2.5 mg/kg) intraperitoneally every 3 days for a total of 3 doses. Tumor volumes were measured every 3 days for up to 3 weeks. Tumor growth curves in the different groups were generated. Each data point in the growth curve represents the mean of 7 determinations ± SE. *P < 0.05; **P < 0.01; ***P < 0.001. (c and d) Apoptotic GFP-WT cells were measured by TUNEL assay in tumor sections. The histogram represents a summary data.

To prove that drug-resistant cells confer a humoral mediated cytoprotective effect on drug-sensitive cells *in vivo*, we assessed the toxicity of doxorubicin in SCID mice bearing xenografts with varying ratios of drug-resistant and drug-sensitive neuroblastoma cells. Mice were injected with 10^6^ mixed SK-N-SH GFP-WT cells and SK-N-SH DoxR (~100 times more resistant than parental drug-sensitive cells) in ratios from 1 : 0 to 1 : 4. When the tumors became palpable, mice received three intra-peritoneal drug injections of doxorubicin (2.5 mg/kg) every 3 to 4 days thereafter. Tumor measurements were made with a caliper three times weekly for 3 to 4 weeks and converted to tumor volume by using the formula *W* × *L* ^2^/2.  Figure 6(a) shows that injections with GFP-WT : DoxR ratios of 1 : 2 and 1 : 4 produced larger tumors than the control (SK-N-SH/GFP cells alone, 1 : 0) and 1 : 1 ratio injections. A TUNEL assay was also performed on these tumor specimens to assess for the degree of apoptosis present (Figure 6(b)). As the ratio of SK-N-SH DoxR to SK-N-SH GFP-WT increased, the percent of apoptotic cells in the GFP cells decreased under UV florescence microscopy. These data confirm that in the *in vivo* tumor microenvironment, doxorubicin resistant cells confer a cytoprotective (antiapoptosis) effect on neighboring drug-sensitive cells which allows them to grow despite drug treatment. In order to confirm that MK was the agent responsible for this cytoprotective effect *in vivo*, these experiments were repeated injecting various ratios of SK-N-SH GFP-WT and MK transfected (SK-N-SH HMK) cells subcutaneously in nude mice with similar results (Figure 6(c)).

### 3.5. Midkine Expression Is Enriched in Human Tissue Samples after Treatment

**Figure 7:**
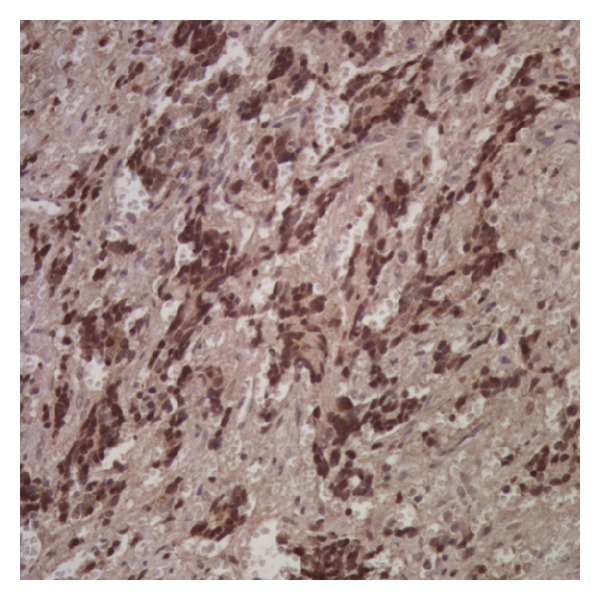
Immunohistochemistry for midkine in human neuroblastoma tissue samples. Prechemotherapy and postchemotherapy patient neuroblastoma biopsy samples were collected and stained for midkine. Midkine staining was scored using a 0 (negative), 1+ (weak staining), 2+ (moderate staining), and 3+ (strong staining) scale on a blinded basis. Midkine staining was then correlated with tumor stage, N-MYC amplification status, histology (favorable or unfavorable), and survival. Biopsy scores were also evaluated for a change in midkine staining prechemotherapy to postchemotherapy. Shown here is a sample neuroblastoma histology slide stained for midkine.

MK expression has previously been implicated as a poor prognostic indicator in oral squamous cell carcinoma [11], neuroblastoma [12, 13], and bladder carcinoma [14]. We sought to determine whether a correlation exists between MK expression and prognostic indicators for neuroblastoma by evaluating the expression of MK in human neuroblastoma tissue samples (Figure 7 and Table 1). After nuclear and cytoplasmic scoring, we found no correlation, in either pretreatment or posttreatment specimens, between MK expression and neuroblastoma stage, N-MYC status, histology (favorable versus unfavorable), age <1 year, or survival. We also compared the expression of MK in pretreatment specimens with posttreatment specimens with the hypothesis that MK expressing, drug-resistant tumor cells would be enriched in posttreatment specimens (Table 2). Histologic scoring revealed that MK expression was enriched in the cytoplasm of posttreatment specimens when compared to pretreatment specimens (*P* < 0.001). These data show that MK is enriched in the cytoplasm of posttreatment tumor specimens, although its expression may not correlate with survival or with prognostic indicators.

## 4. Discussion

In 1988, the Muramatsu group first described midkine as a 13–15 KD heparin binding polypeptide present in embryonal carcinoma cells which acts to enhance neuronal cell survival and stimulate neurite extrusion [19]. Midkine has subsequently been found to be overexpressed in a number of malignant tumors [8–10]. Midkine has been found to exert a survival function in a variety of cell types and against various stimuli, suggesting a potential role in drug resistance [16, 20].

Our laboratory has demonstrated that an intercellular crosstalk occurs between cells that have acquired a drug resistance phenotype and neighboring drug-sensitive tumor cells [6]. Communication between drug-resistant cells by secretion of survival factors, such as midkine, is postulated to protect cells in the surrounding environment and enable survival of the tumor as a whole. We previously showed that wild type human neuroblastoma cells treated with conditioned medium from doxorubicin resistant cells become more resistant to doxorubicin [6]. However, although midkine was found to be upregulated in this conditioned media, it remained possible that any number of other secreted survival factors could have contributed to the conferred resistance. To verify this result and rule out other growth and/or survival factors which may also contribute to the observed effect, a co-culture system was used in the present study. The benefit of this system is that it allowed drug-sensitive and drug-resistant cells to grow in separate compartments without physical contact but permitted the diffusion of secreted molecules or drugs from one compartment to the other by traversing the membrane. The results presented here demonstrate that midkine transfected cells have almost the same ability for cellular protection as those of drug-resistant cells through a Pgp independent mechanism. This serves as direct evidence of crosstalk between drug-resistant-and drug-sensitive cells with midkine identified as being the protein involved in this mechanism, allowing for conferred drug resistance.

Our *in vitro *and *in vivo *studies demonstrated a significant cytoprotective effect of doxorubicin resistant cells or midkine expressing cells on wild type cells. This effect was abolished when midkine expression was downregulated in drug-resistant cells using siRNA. In accordance with our results, Takei et al. reported that inhibition of midkine secretion and expression using siRNA in prostate cancer PC-3 cells leads to significant suppression of tumor growth in a xenograft model [21]. Dai et al. significantly suppressed the growth of hepatocellular carcinoma *in vitro* and *in vivo* using nanoliposomes packaged with antisense phosphorothioate oligonucleotide targeting midkine (MK-ASODN) [22]. These data suggest that inhibition of midkine may constitute a useful therapeutic target.

Midkine expression is usually low in normal human adult tissues. Wilms' tumor, neuroblastoma, esophageal, pancreatic, lung, and breast cancers have all been shown to have enriched MK expression [8–10]. In addition, increased midkine expression is associated with poor prognosis in oral squamous cell carcinoma [11], neuroblastoma [12, 13], and bladder carcinoma [14]. Ikematsu et al. demonstrated that plasma midkine levels in neuroblastoma patients correlated significantly with prognostic indicators in neuroblastoma including MYCN amplification, ploidy, tumor stage, and age [13]. We were able to demonstrate an increase in cytoplasmic midkine staining in postchemotherapy biopsy specimens relative to prechemotherapy specimens. These data suggest that midkine expression is induced or acquired during the acquisition of drug resistance and support our *in vitro *finding of increased midkine expression in neuroblastoma cells which acquire drug resistance through long-term drug exposure. Our histopathologic analysis did not demonstrate a correlation of tumor midkine expression levels with prognostic indicators. This finding however may be due to lack of power with the small sample size of biopsy specimens available for these investigations. It may also be due to MK primary role as a secreted factor and therefore not present in the immunohistochemistry results.

The exact mechanism by which midkine mediates drug resistance remains poorly understood, but it is postulated that midkine has anti-apoptotic (or antiautophagy) [23–25] and proangiogenic activity [9, 26] promoting survival in human tumors. Midkine was found to protect Wilms' tumor cells from cisplatin-induced apoptosis through *Bcl-2* enhanced expression [16]. In human pediatric rhabdoid tumor G401 cells, midkine activates the anti-apoptotic pathway mediated by *STAT1* and the Janus-activated kinases (*JAKs*) [27]. Midkine has been shown to activate the survival pathway mediated by *Akt* in cultured neurons leading to a neuroprotective effect [23]. Furthermore, You et al. reported that midkine is a NF-*κ*B-inducible gene which supports prostate cancer cell survival [28]. Recently, Güngör et al. [29] identified an interaction between the Notch-2 receptor and midkine in pancreatic ductal adenocarcinoma (PDAC) cells. They found that MK-Notch-2 interaction activated Notch signaling, upregulating *NF-*κ*B* inducing epithelial-mesenchymal transition (EMT), and increased chemoresistance. 

Perhaps the most intriguing questions regarding the anti-apoptotic activity of midkine and other heparin binding growth factors reside in determining potential sites of action in the cell. Because midkine is secreted from drug-resistant cells, the neighboring drug-sensitive cells must express a receptor for midkine as well as the related signaling pathway. By virtue of its apparent lack of specificity, midkine may bind nonspecifically to and activate various receptors, leading to activation of more than one survival pathway and thus increasing its chances to mediate cytoprotection. At least four receptors, namely, N-syndecan [30], RPTPz [31], ALK [24, 32] and LRP1 [33], have been proposed to mediate the biological activities of midkine [33], and extensive studies have been undertaken investigating the potential roles of these pathways. 

In summary, the evidence presented in this study confirms the existence of an intercellular cytoprotective signal within drug-resistant tumors which allows phenotypically drug-resistant tumor cells to protect nearby otherwise drug sensitive cells. This humoral effect appears to be mediated primarily by midkine, allowing protection of neighboring cells from drug-induced killing. Future studies identifying the pertinent midkine receptors and downstream signaling pathways may identify novel tumor markers or therapeutic targets.

## Figures and Tables

**Table 1:** Correlation of midkine staining in human tissues with prognostic indicators.

	Nuclear score				Cytoplasmic score			
	Prechemo	*P*	Postchemo	*P*	Prechemo	*P*	Postchemo	*P*
Stage		0.46		0.16		0.23		0.22
I	3.0 ± 0.0		3.0 ± 0.0		1.3 ± 0.9		0	
II	3.0 ± 0.0		3.0 ± 0.0		0.0 ± 0.0		3.0 ± 0.0	
III	1.8 ± 1.1		2.0 ± 1.0		0.0 ± 0.0		2.8 ± 0.4	
IV	2.1 ± 1.2		1.0 ± 1.0		1.0 ± 1.1		2.0 ± 1.4	
N-MYC Status		0.86		0.41		0.48		0.26
Nonamplified	2.2 ± 1.2		1.8 ± 1.2		0.8 ± 1.1		1.8 ± 1.3	
Amplified	2.3 ± 0.9		1.0 ± 0.0		1.2 ± 1.2		3 ± 0	
Histology		0.49		0.34		0.77		0.51
Favorable	2.4 ± 0.8		2.0 ± 1.0		0.8 ± 1.0		3.0 ± 0.0	
Unfavorable	1.9 ± 1.4		1.3 ± 1.1		1.0 ± 1.2		2.3 ± 1.3	
Age		0.17		0.20		0.22		0.90
<1 year	1.9 ± 1.4		1.3 ± 1.2		1.2 ± 1.2		2.1 ± 1.4	
>1 year	2.6 ± 0.7		2.2 ± 1.0		0.6 ± 0.9		2.2 ± 1.2	
Survival status		0.64		0.79		0.33		1
Survivor	2.1 ± 1.2		1.5 ± 1.1		0.8 ± 1.1		2.3 ± 1.1	
Nonsurvivor	2.4 ± 1.2		1.3 ± 1.1		1.4 ± 1.2		2.3 ± 1.3	

**Table 2:** Midkine expression prechemotherapy compared to postchemotherapy.

	Nuclear Score			Cytoplasmic score		
	Pre-chemo	Post-chemo	*P*	Pre-chemo	Post-chemo	*P*
All biopsies*	2.3 ± 1.1	1.7 ± 1.2	0.15	0.9 ± 1.1	2.2 ± 1.3	0.005
Matched biopsies**	2.1 ± 1.3	1.9 ± 1.3	0.74	0.4 ± 0.8	2.3 ± 1.2	<0.001

*Comparison based on 20 prechemotherapy biopsies and 12 postchemotherapy biopsies.

**Comparison based only on the 10 patients with matched pre- and postchemotherapy biopsies available for analysis.
